# Flexible, Freely Available Stochastic Individual Contact Model for Exploring COVID-19 Intervention and Control Strategies: Development and Simulation

**DOI:** 10.2196/18965

**Published:** 2020-09-18

**Authors:** Timothy Churches, Louisa Jorm

**Affiliations:** 1 Ingham Institute for Applied Medical Research South Western Sydney Clinical School, Faculty of Medicine University of New South Wales Sydney Liverpool Australia; 2 Centre for Big Data Research in Health Faculty of Medicine University of New South Wales Sydney Randwick Australia

**Keywords:** COVID-19, epidemic curve, infection dynamics, public health interventions

## Abstract

**Background:**

Throughout March 2020, leaders in countries across the world were making crucial decisions about how and when to implement public health interventions to combat the coronavirus disease (COVID-19). They urgently needed tools to help them to explore what will work best in their specific circumstances of epidemic size and spread, and feasible intervention scenarios.

**Objective:**

We sought to rapidly develop a flexible, freely available simulation model for use by modelers and researchers to allow investigation of how various public health interventions implemented at various time points might change the shape of the COVID-19 epidemic curve.

**Methods:**

“COVOID” (COVID-19 Open-Source Infection Dynamics) is a stochastic individual contact model (ICM), which extends the ICMs provided by the open-source EpiModel package for the R statistical computing environment. To demonstrate its use and inform urgent decisions on March 30, 2020, we modeled similar intervention scenarios to those reported by other investigators using various model types, as well as novel scenarios. The scenarios involved isolation of cases, moderate social distancing, and stricter population “lockdowns” enacted over varying time periods in a hypothetical population of 100,000 people. On April 30, 2020, we simulated the epidemic curve for the three contiguous local areas (population 287,344) in eastern Sydney, Australia that recorded 5.3% of Australian cases of COVID-19 through to April 30, 2020, under five different intervention scenarios and compared the modeled predictions with the observed epidemic curve for these areas.

**Results:**

COVOID allocates each member of a population to one of seven compartments. The number of times individuals in the various compartments interact with each other and their probability of transmitting infection at each interaction can be varied to simulate the effects of interventions. Using COVOID on March 30, 2020, we were able to replicate the epidemic response patterns to specific social distancing intervention scenarios reported by others. The simulated curve for three local areas of Sydney from March 1 to April 30, 2020, was similar to the observed epidemic curve in terms of peak numbers of cases, total numbers of cases, and duration under a scenario representing the public health measures that were actually enacted, including case isolation and ramp-up of testing and social distancing measures.

**Conclusions:**

COVOID allows rapid modeling of many potential intervention scenarios, can be tailored to diverse settings, and requires only standard computing infrastructure. It replicates the epidemic curves produced by other models that require highly detailed population-level data, and its predicted epidemic curve, using parameters simulating the public health measures that were enacted, was similar in form to that actually observed in Sydney, Australia. Our team and collaborators are currently developing an extended open-source COVOID package comprising of a suite of tools to explore intervention scenarios using several categories of models.

## Introduction

March 2020 was a critical time in the global coronavirus disease (COVID-19) pandemic, when political leaders and policy makers were making crucial decisions that would shape the lives and futures of people and communities. “Flattening the curve” had become a rallying cry in the fight against COVID-19, popularized by media outlets and leaders worldwide. However, the ubiquitous COVID-19 “flattening the curve” infographic [1] can be traced back to a purely conceptual diagram in a 2007 US Centers for Disease Control and Prevention report recommending strategies for pandemic influenza mitigation [2]. It was essential that political leaders and their advisers had ready access to more sophisticated mathematical and computational tools to allow them to explore quickly and iteratively how implementing various public health interventions would potentially change the shape of the COVID-19 epidemic curve in their settings.

Stochastic individual contact models (ICMs), also known as individual-based or agent-based models, are increasingly used for epidemic simulation modeling. These models represent individual units in the population and the contacts between them as discrete events and capture the stochasticity seen in real-world disease outbreaks. Compared with more traditional deterministic compartmental models (DCMs), which are based on systems of differential equations for the movement of the population through discrete states at specified rates, they may produce more realistic results, especially in situations where microepidemics emerge at city and community levels [3].

On March 30, 2020, ICMs for COVID-19 had recently been reported for the United Kingdom, the United States [4], and Australia [5], adapted from existing models for pandemic influenza. These use whole-of-population census data and model contacts between individuals in the population within households, schools, workplaces, and in the wider community. The UK model appears to have been influential in driving a turnaround in the COVID-19 response strategy in that nation [6]. The Australian model highlighted the potential for the virus to spread virtually unchecked unless there were high levels of compliance with social distancing measures [7].

Given the enormous consequences of decisions about public health interventions that were being made at that time, it was highly desirable to independently assess the robustness of these (not yet peer reviewed) ICMs. However, the software code for these models has not been made publicly available, limiting scrutiny of their underlying structure and making it impossible to exactly replicate their findings or test sensitivity to alternative assumptions.

Furthermore, the ICMs reported on March 30, 2020, reflected the circumstances of high-income western nations. Their findings may not be applicable in countries and communities that have substantially different demography, social network structures, education and health systems, workplaces, and community resources. Replicating them rapidly in other settings is challenging because they require the ready availability of detailed population-level data. Furthermore, running them requires access to high-performance computing, which is not feasible in many settings.

Our objective is to develop a flexible, freely available COVID-19 ICM simulation model for use by modelers and researchers that can be tailored to diverse settings and run using standard desktop or laptop computing hardware. Importantly, given the quickly evolving situation worldwide, we sought to build a model that permitted highly flexible definitions of intervention strategies that more closely reflect the real world, in which epidemic control measures tend to take time to implement, often less completely than hoped, and that cannot be and are not sustained indefinitely.

## Methods

### Model Building

**Figure 1:**
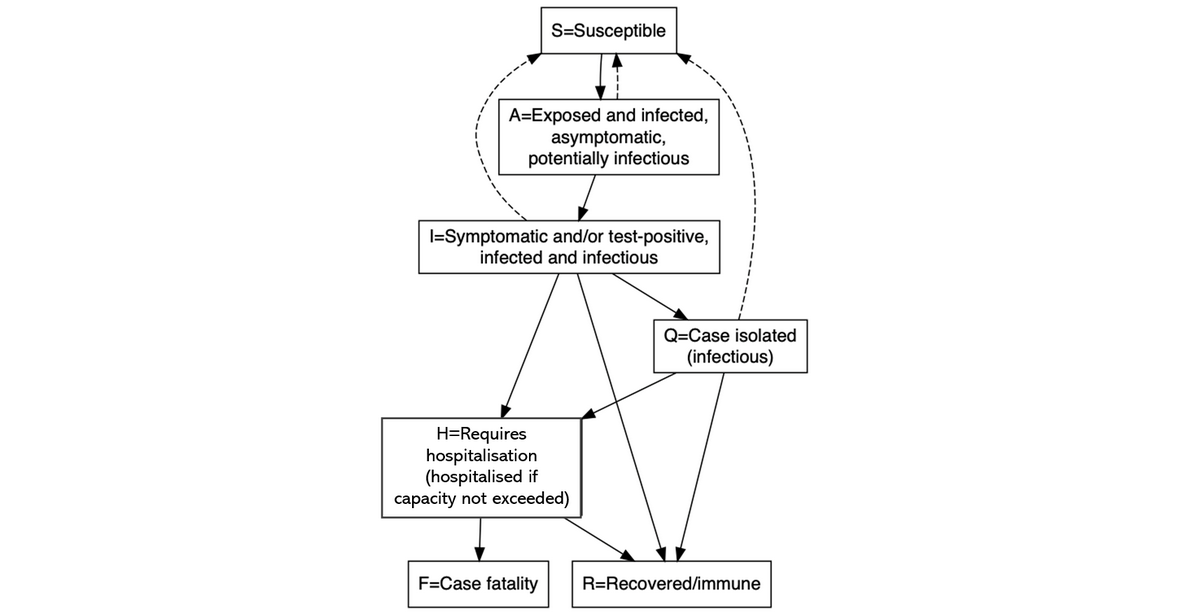
Structure of the COVID-19 Open-source Infection Dynamics stochastic individual contact model. The dashed arrows represent interpersonal interactions through which transmission of infection may occur. The solid arrows indicate possible transitions between compartments. COVID-19: coronavirus disease.

“COVOID” (COVID-19 Open-Source Infection Dynamics) is a stochastic ICM that we constructed by extending the peer-reviewed [8] open-source EpiModel package [9] for the widely-used, open-source R statistical computing environment (R Foundation for Statistical Computing) [10]. Our model extensions allocate each member of a hypothetical population to one of seven compartments (Figure 1). We have replaced the traditional E (exposed) compartment as used in susceptible-exposed-infectious-recovered (SEIR) models, with an A (infected and asymptomatic) compartment, representing infected, asymptomatic individuals who are nonetheless potentially infectious. Additional compartments, representing symptomatic or individuals who have tested positive in self-isolation (isolated [Q]) and an infected individual that requires hospitalization (H) were also added, as well as a compartment for deaths due to COVID-19 (F) as distinct from deaths due to other causes, which together with emigration are handled by a separate demographic removal process.

At each 1-day time step of the simulation, individuals randomly encounter and are exposed to other individuals in the population. The intensity of this population mixing is controlled by an act rate parameter specific to each of the infectious compartments (A, infected and infectious [I], and Q), with each “act” representing an opportunity for disease transmission or at least those “acts” between susceptible individuals and infectious individuals. Recovered individuals are no longer infectious and are assumed to be immune from further reinfection; thus, their interactions do not result in infections, nor do interactions between pairs of susceptible individuals nor pairs of infectious individuals; only the interactions between susceptible and infectious individuals may give rise to new infections. However, not every such opportunity for disease transmission will result in actual disease transmission. The probability of transmission at each interaction is controlled by an infection probability parameter, also specific to each of the infectious compartments (A, I, and Q).

Thus, the interventions are simulated by varying the act rate parameter (equivalent to social distancing in the population) and the infection probability parameter (equivalent to increased practice of hygiene measures such as hand washing, use of hand sanitizers, not touching one’s face, and mask wearing by the infectious). The act rate and infection probability for the isolated compartment (Q) are set to lower levels than for the asymptomatic infected and infectious (A) and symptomatic or test-positive infected and infectious (I) compartments. Other parameters can also be changed, as a function of time (so they can be ramped up and ramped down or pulsed, as required) to simulate public health interventions, such as changes to the rate at which individuals in the symptomatic or test-positive I compartment enter the isolation Q compartment.

### Intervention Scenarios Modeled for March 30, 2020

We used COVOID to model intervention scenarios in a hypothetical population of 100,000 people. A baseline case assuming no interventions were established using parameters based on values in the literature. Interventions were then simulated by varying the number of times individuals in the various compartments interact with each other (the act rate for each of the infectious compartments A, I, and Q).

The baseline case assumes 3 symptomatic infected individuals (compartment I) at day 1, plus 4 asymptomatic but infected individuals (compartment A). The initial value for the I compartment was chosen heuristically, and we assumed that 60% of infected individuals were asymptomatic based on the findings in Japanese citizens repatriated from Wuhan, as reported by Mizumoto et al [11], which were the best estimates available at the time. Other parameters were based on those used by Constantino, Heslop, and Macintyre [12], which were in turn based on the best estimates available in the preprint literature at the time. We specified an average of 8.5 interpersonal interactions per day, 5% probability of infection following interactions with symptomatic infectious individuals (I compartment), 2% probability of infection following interactions with asymptomatic infectious individuals (A compartment), and just 3% of symptomatic individuals (I compartment) self-isolate on each day of illness, with subsequently 2.5 personal interactions per day while in self-isolation. Hospital capacity is set at 1148 beds, approximating the Australian average of 3.8 beds per 1000 population [13], and the rate of fatalities in those requiring hospitalization (H compartment) is doubled for the prevalent cases above this capacity limit who require hospitalization.

The parameters for both the initial and subsequent baseline models are shown in Table 1.

**Table 1:** Parameters used for baseline models.

Parameter	Value	Description	Rationale
Start date	March 1, 2020	Day 1 of simulation	Beginning of sustained community transmission in NSW^a^, Australia
Initial S^b^ compartment (March 30 models), n	100,000	Susceptible population at day 1	Hypothetical population used for initial March 30, 2020 models
Initial S compartment (April 30 models), n	287,337	Susceptible population at day 1	Population of Waverley, Woolahra, and Randwick local government areas in eastern Sydney [14]
Initial A^c^ compartment, n	4	Infected but asymptomatic persons at day 1	Assuming 60% of infected persons are asymptomatic based on Mizumoto et al [11]
Initial I^d^ compartment, n	3	Infected but symptomatic or persons who are test-positive at day 1	Number of detected cases in modeled population in 3 weeks prior to start date
Q^e^, R^f^, H^g^, and F^h^ compartments, n	0	Other compartments at day 1	Assumed empty at start
*Act rate* (social contact rate) per day for A and I compartments, n	8.5	Number of social contacts with potential for infection per day per individual	Based on average daily contact rates given in Table 1 of Eames et al
*Act rate* (social contact rate) per day for Q compartment, n	1.5	As above	Adapted from reduction in transmission for those in isolation or quarantine used by Constantino et al [12]
*Infection probability*, n	0.05 for I compartment, 0.02 for A and Q compartments	Probability of transmitting infection at each encounter as defined by *act rate*	No published values for COVID-19^i^ found in literature, heuristic values based on discussions with subject matter experts
*Isolation rate* per day, n	0.033	Proportion of symptomatic people putting themselves into self-isolation per day of symptoms, in absence of public health information encouraging then to do so	No values found in literature, heuristic value based on discussions with subject matter experts
*Progression rate*	Discrete Weibull distribution, mean 5, shape 1.5	Distribution of time in A compartment, equivalent to the incubation time	Adapted from values used by Constantino et al [12]
*Hospitalization rate* per day, n	0.01	Crude (non–age-specific) proportion of people in I compartment that require hospitalization per day in compartment	Adapted from values used by Constantino et al [12]
*Discharge rate* per day, n	0.05	Proportion of persons in H compartment who are discharged from needing hospital care each day	Reciprocal of mean length of stay, based on values used by Constantino et al [12]
*Recovery rate* per day, n	0.05	Proportion recovering each day, based on reciprocal of mean duration of illness of 20 days	Based on value used by Constantino et al [12]
*Fatality base rate* per day, n	0.02	Proportion of persons in H compartment if number is less than or equal to hospital capacity who die each day	Based on mean death rates used by Constantino et al [12]
*Fatality above capacity rate* per day, n	0.04	Proportion of persons in H compartment in excess of hospital capacity who die each day	Heuristic value, no relevant COVID-19 data relating to this found in literature

^a^NSW: New South Wales.

^b^S: susceptible.

^c^A: infected and asymptomatic.

^d^I: infected and infectious.

^e^Q: isolated.

^f^R: recovered.

^g^H: requires hospitalization.

^h^F: deaths due to COVID-19.

^i^COVID-19: coronavirus disease.

Chang et al [5] used a highly detailed agent-based model for the entire Australian population, originally developed to investigate influenza transmission, to investigate the effect of 90-day periods of reduced social mixing (social distancing) in which 90%, 80%, and 70% of the population were assumed to be instantaneously compliant, compared to their baseline model.

Using the baseline parameters shown in Table 1, we investigated the same intervention scenarios, as well as 60% and 50% compliance levels, by using weighted means of compliant and noncompliant act rate parameters. The scenarios are listed in Table 2. Because we were simulating in a hypothetical population of only 100,000, resulting in faster spread than would occur in the full Australian population of 25 million, we initiated the social distancing interventions at 15 days, rather than at 45 days as done by Chang et al [5].

**Table 2:** Scenarios modeled for March 30, 2020.

Scenario	Description
Scenario 01	Starting at day 15 (March 15, 2020), instantaneous imposition of 90% social distancing for 90 days, then instantaneous reversion to baseline social contact rate
Scenario 02	Starting at day 15 (March 15, 2020), instantaneous imposition of 80% social distancing for 90 days, then instantaneous reversion to baseline social contact rate
Scenario 03	Starting at day 15 (March 15, 2020), instantaneous imposition of 70% social distancing for 90 days, then instantaneous reversion to baseline social contact rate
Scenario 04	Starting at day 15 (March 15, 2020), instantaneous imposition of 60% social distancing for 90 days, then instantaneous reversion to baseline social contact rate
Scenario 05	Starting at day 15 (March 15, 2020), instantaneous imposition of 50% social distancing for 90 days, then instantaneous reversion to baseline social contact rate

### Comparison of Modeled vs Observed Epidemic Curves in Sydney, Australia for April 30, 2020

The first cases of COVID-19 were reported in Australia on January 24, 2020. The island of Australia has a vast geography and sparse population, and has limited border entry points. The city of Sydney, capital of the state of New South Wales (NSW), is the major entry point for international travelers. As of April 30, 2020, 358 out of 6746 (5.3%) of Australia’s recorded locally acquired cases of COVID-19 were among residents of three contiguous local government areas of Sydney: Randwick, Waverley, and Woollahra, with a combined population of 287,344 [14]. As of April 30, 55% of cases in Woollahra, Waverley, and Randwick were locally acquired [14], and they were among 13 “high risk” local government areas in NSW where immediate testing of all symptomatic people was encouraged from April 6, 2020. To compare scenarios modeled using COVOID with observed Australian data from the COVID-19 epidemic, we ran simulations for incident cases in the combined population of these three local areas, where it could be assumed that the population had ample opportunities for mixing and exposure to the virus.

A staged series of public health measures were enacted in Australia from February 1, 2020, summarized as they applied in the state of NSW in Table 3.

**Table 3:** Coronavirus disease public health measures enacted in the state of New South Wales, Australia, February 1 to April 30, 2020.

Date (2020)	Public health measures enacted
February 1	Borders closed to all nonresidents and non-Australian citizens who had left or transited through Mainland China
March 16	Outdoor events with more than 500 attendees banned
March 17	Self-isolation (14 days) for overseas travelers
March 20	Borders closed to all nonresidents and non-Australian citizens
March 21	Social distancing rule of 4 square meters per person in any enclosed space
March 23	Pubs, clubs, gyms, indoor sporting venues, entertainment venues closed, and food outlets restricted to takeaway or delivery
March 26	Closures extended to include places such as personal services, arcades, brothels, galleries, museums, swimming pools, community facilities, libraries, gambling venues, and markets
March 29	Public gatherings limited to two people; people only to leave their houses for: shopping for essentials, medical or compassionate needs, exercise in compliance with the public gathering restriction, or work or education purposes.
March 30	Mandatory isolation in hotels for travelers
April 28	Gradual easing of restrictions commences

To compare our simulations with observed incidence data, we chose a starting date for our simulations of March 1, 2020, 15 days prior to the gradual ramp-up of social distancing measures in NSW. At that date, 3 cases had been recorded in the three eastern Sydney local government areas used for our model; thus, we initialized the model with 3 persons in the I compartment. As previously noted, we assumed approximately 60% of infections were asymptomatic and thus also initialized the model with 4 persons in the A compartment. Other parameters were also as per the baseline model previously described.

Using this baseline model for eastern Sydney, we then modeled several scenarios to explore the effect of various intervention strategies on the fit of our baseline model to the observed data. The scenarios are described in Table 4. In particular, scenarios 08 and 10 were intended to mimic the actual interventions that had occurred in Sydney on April 30, 2020.

**Table 4:** Scenarios modeled for April 30, 2020.

Scenario	Description
Scenario 06	Starting at day 1 (March 1, 2020), linear ramp up of self-isolation rate (per day) from 3.3% to 33% over a 15-day period, then hold at 33% indefinitely
Scenario 07	Isolation rates per scenario 06, plus a moderate increase in social distancing to 50% starting at day 15 (March 15, 2020) by linearly ramping the *act rate* per day down from 8.5 to 4.75 over a 15-day period (through to March 30, 2020), then maintaining social distancing at 50% (*act rate*=4.65) for a further 45 days, then reverting immediately to no social distancing (*act rate*=8.5 per day)
Scenario 08	Isolation rates as per scenario 06, plus a substantial increase in social distancing to 80% starting at day 15 (March 15, 2020) by linearly ramping the *act rate* per day down from 8.5 to 2.5 over a 15-day period (through to March 30, 2020), then maintaining social distancing at 80% (*act rate*=2.5) for a further 30 days, then reverting immediately to 50% social distancing (*act rate*=4.75 per day) on an ongoing basis
Scenario 09	Isolation rates as per scenario 06 plus a substantial increase in social distancing to 80% starting at day 15 (March 15, 2020) by linearly ramping the *act rate* per day down from 8.5 to 2.5 over a 15-day period (through to March 30, 2020), then maintaining social distancing at 80% (*act rate*=2.5) for a further 30 days, then slowly reverting to no social distancing (*act rate*=8.5 per day) over the subsequent 90-day period
Scenario 10	As per scenario 09 but, immediately following the full “lockdown” period between March 30 and April 30, 2020, there is a linear increase of the isolation rate (per day) from 33% to 66% over a 30-day period through to May 28, 2020, with subsequent maintenance of self-isolation with high compliance (66% per day) on an ongoing basis.

We compared the epidemic curves simulated by COVOID with reported data for locally acquired new cases for Randwick, Waverley, and Woollahra for the period March 1, 2020, to April 30, 2020 [15], by examining modeled and observed daily peak and total numbers of incident cases.

### Software and Code

COVOID is implemented on top of EpiModel v1.8 [9] running on R version 3.6.1 [10]. The COVOID model is described in more detail in the technical blog of the first author [16], and all the code used for the simulations reported in this paper is available at [17] and [18].

## Results

### Computing Resources

The twelve simulations reported in this paper were each run eight times and the results averaged, taking approximately 60 minutes to complete when running in parallel on an eight-core Intel central processing unit (CPU). The same set of simulations for a population of 1,000,000 were also run successfully on the same hardware, taking approximately 3 hours and using less than 16GB of RAM, suggesting that run times scale as a low-order power of the population size. Running on 1, 2, 4, or 8 CPU cores resulted in near-linear reductions in total run times, which was expected given that each simulation run is independent. Scaling to use more CPU cores is automatic, and near real time response would be possible on suitably sized cloud computing infrastructure, if required.

### Baseline Model

**Figure 2:**
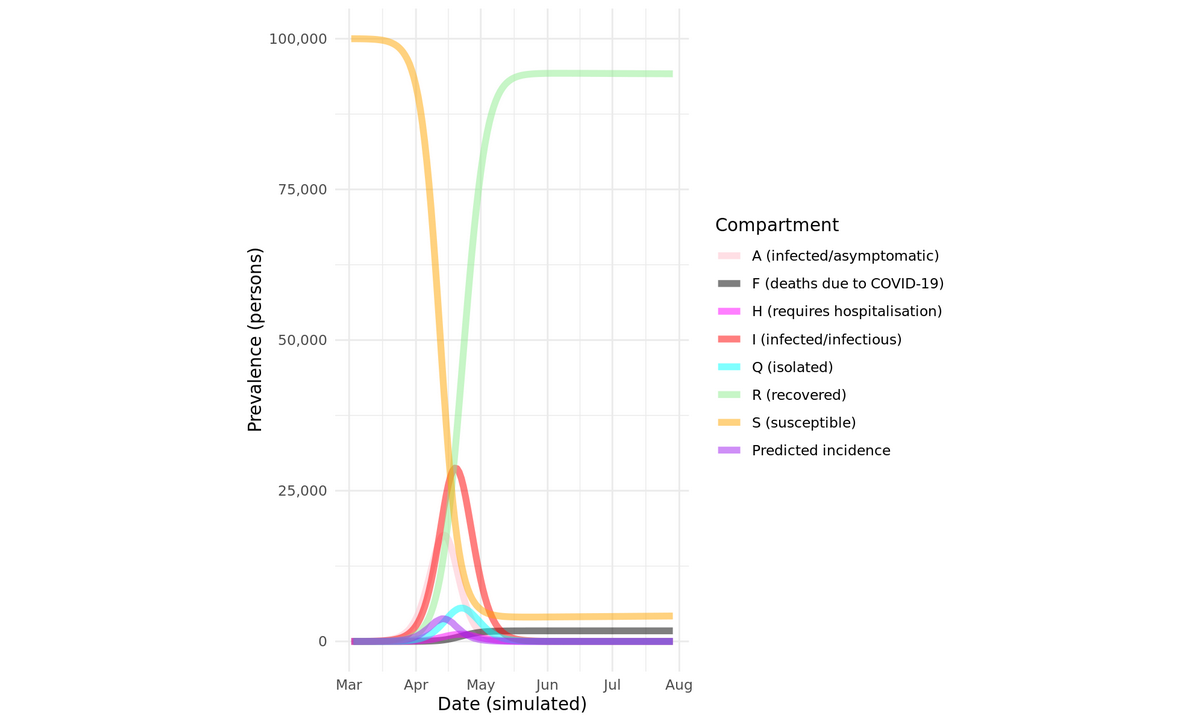
Baseline simulation with hypothetical 100,000 population. COVID-19: coronavirus disease.

The results of the baseline model simulated for a hypothetical population of 100,000 people, without any public health interventions, is shown in Figure 2. Unsurprisingly, nearly 90% of the population are infected within 2 months, with several thousand projected deaths due to COVID-19 infection. These projections are unrealistic because a complete lack of public health intervention (or equivalent spontaneous behavior modification in the population) has not occurred anywhere, but they serve to show that the baseline model produces the expected results.

**Figure 3:**
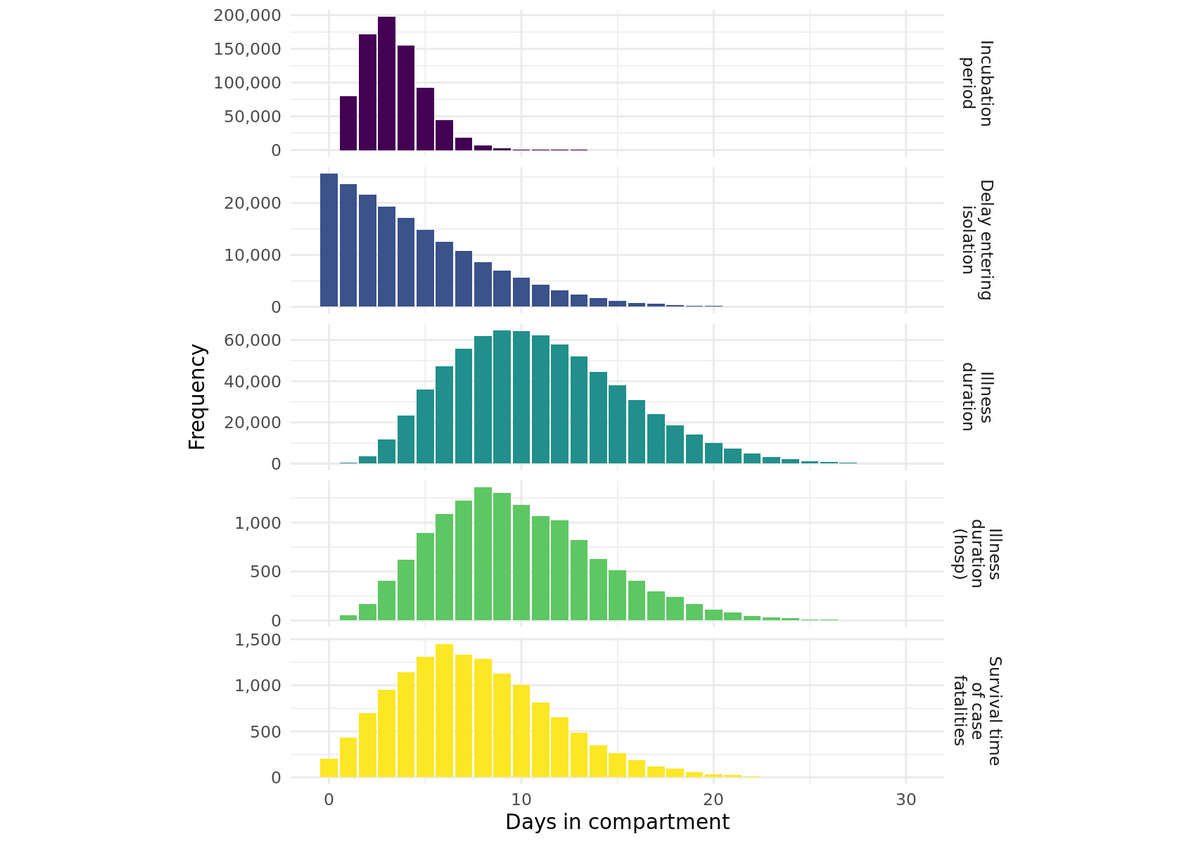
Distributions of time in each compartment in the baseline model. hosp: hospital.

An important but rarely reported aspect of simulation models is the distribution of (simulated) persons in each compartment of the model. This provides additional assurance that flows between compartments reflect known or expected distributions of real-life times in various disease states corresponding to the compartments. The distribution of durations in key model compartments for the baseline model are shown in Figure 3.

### Social Distancing Scenarios With Varying Compliance Modeled for March 30, 2020

**Figure 4:**
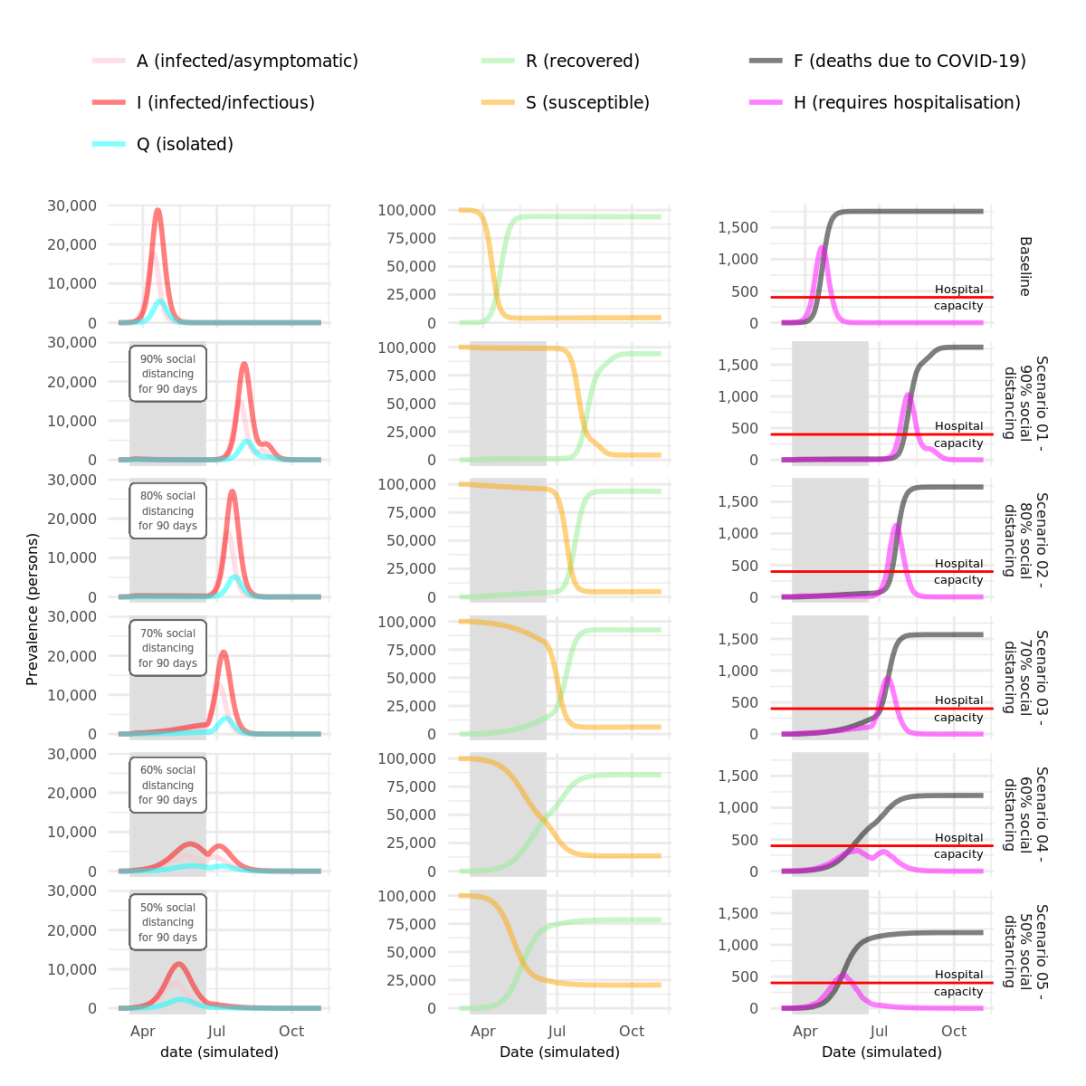
Social distancing scenarios with varying compliance modeled on March 30, 2020. COVID-19: coronavirus disease.

The results of COVOID modeling of 90-day periods of social distancing with instantaneous effect and varying levels of compliance, based on interventions modelled by Chang et al [5], are shown in Figure 4. Social distancing with at least 80% compliance completely suppresses the epidemic for the duration of the intervention, while compliance of 70% still substantially reduces cases and deaths. In each of these scenarios, cases rebound dramatically once social distancing is relaxed, demonstrating that ongoing control measures will be required. These findings are similar overall to those reported by Chang et al [5], noting the differences in time frames due to the different population sizes being modeled.

We modeled two additional scenarios of 60% and 50% compliance with social distancing and found that, although these flatten the epidemic curve compared to the baseline scenario, transmission is not halted, and substantial numbers of cases and deaths occur during the intervention period. In the 50% compliance scenario, hospital capacity is overwhelmed during the intervention period. However, sufficient herd immunity is attained in the 50% social distancing scenario to prevent any second wave of infection after social distancing is relaxed at the expense of considerable morbidity and mortality, and an overwhelmed hospital system while social distancing is in place.

### Comparison of Modeled Interventions vs Observed Epidemic Curves in Sydney, Australia for April 30, 2020

**Figure 5:**
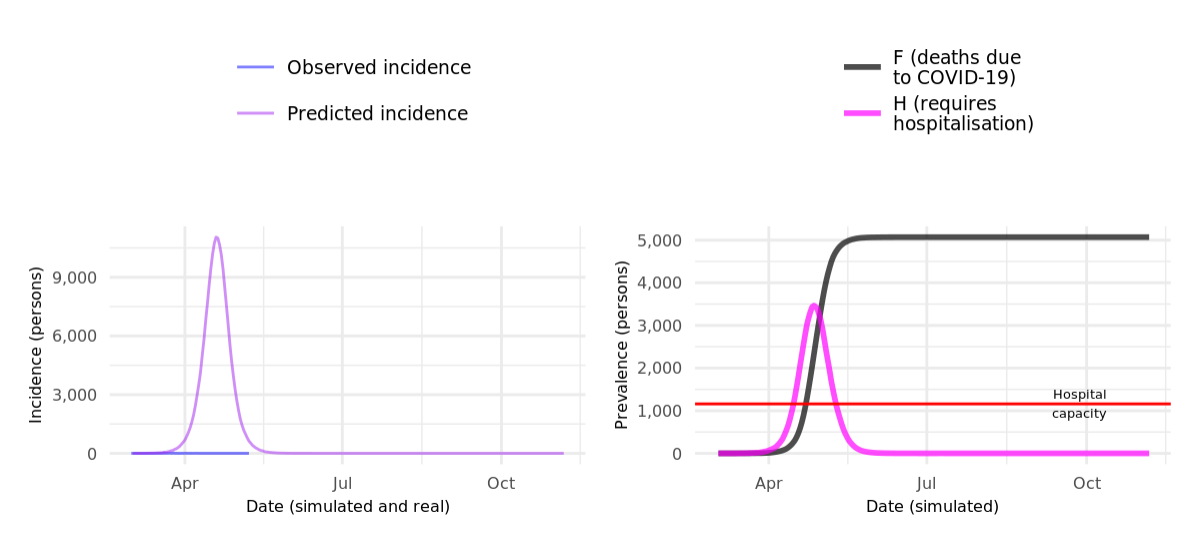
Eastern Sydney baseline simulation, no interventions. COVID-19: coronavirus disease.

**Figure 6:**
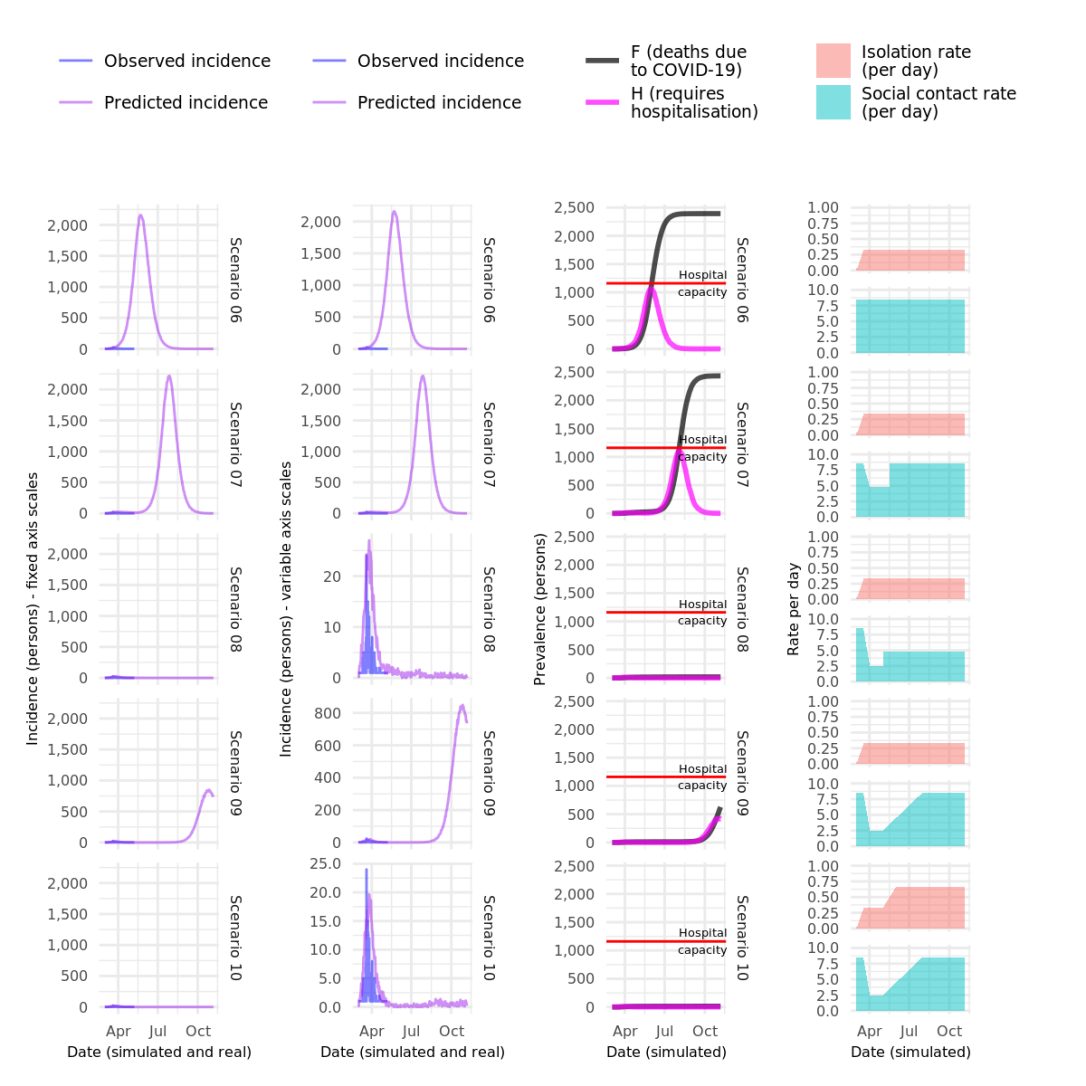
Comparison of modeled vs observed epidemic curves in Sydney, Australia on April 30, 2020. COVID-19: coronavirus disease.

**Figure 7:**
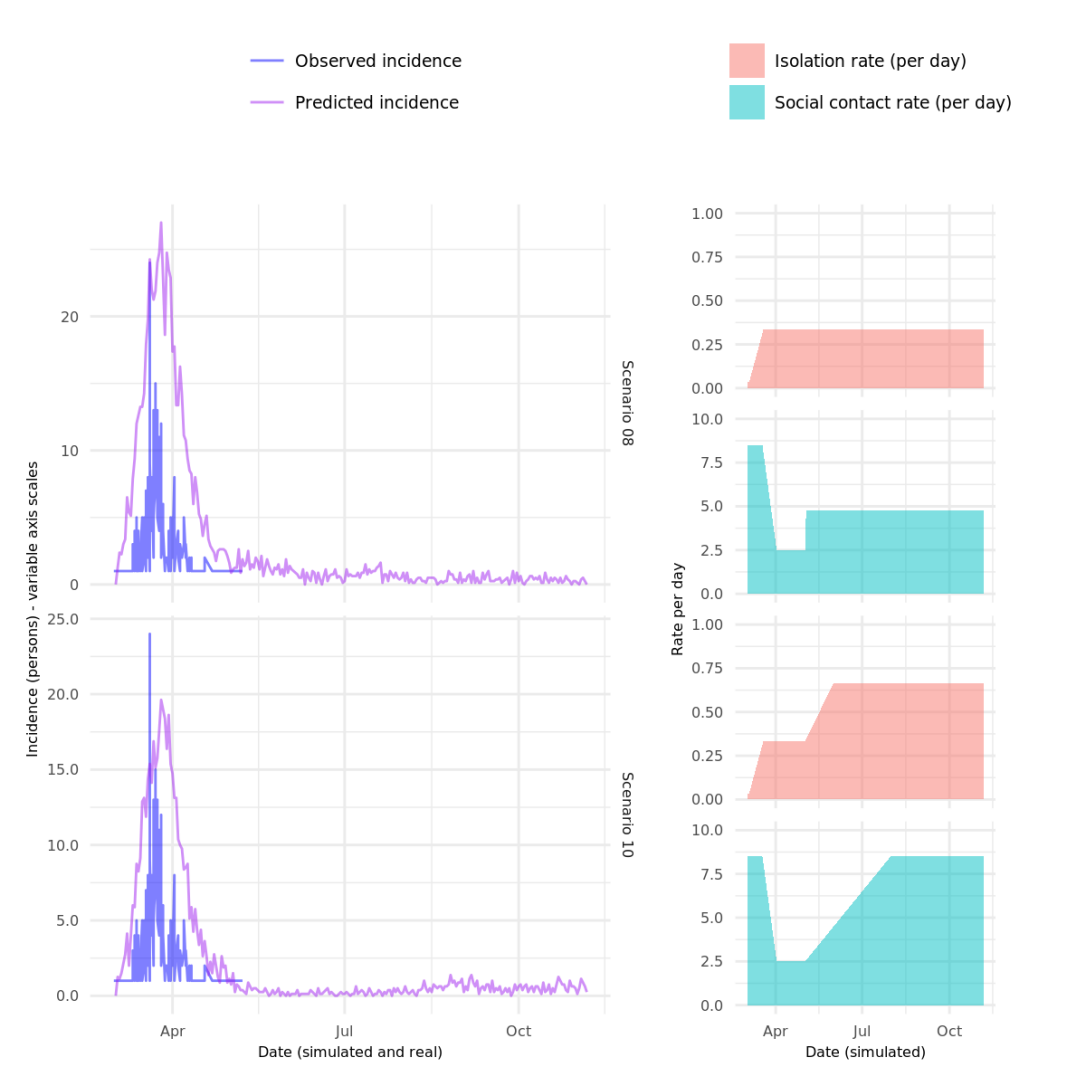
Details for scenarios 08 and 10.

The results of COVOID modeling of the eastern Sydney population, using the same parameters as the baseline model previously shown, are displayed in Figure 5. Unsurprisingly, hospital capacity is quickly exceeded, resulting in a large number of deaths as people die without receiving adequate medical care. However, as can be seen in the left panel of Figure 5, in retrospect, this scenario is also completely unrealistic. The results using various scenarios that approximate public health interventions as they occurred in NSW, Australia during March and April 2020 are shown in Figures 6 and 7. The actual, observed incidence of confirmed COVID-19 infections in the same eastern Sydney population is similarly shown in the left two columns in those figures. Under all scenarios, compared to the baseline simulation, the COVID-19 epidemic curve is substantially flattened and “shrunk” due to case-based interventions, specifically isolation and self-isolation of all symptomatic or test-positive cases with moderate alacrity (33% of cases entering isolation each day post–symptom onset or test result). Under none of the modeled intervention scenarios does the number of cases requiring hospitalization overwhelm assumed hospital capacity, but a significant number of deaths nevertheless occur in several of the scenarios.

Scenario 06 demonstrates that moderate compliance with self-isolation, with no increase in social distancing, substantially dampens the epidemic and reduces deaths by 50%. Scenario 07, which adds 1 month of moderate social distancing (at considerable social and economic cost), shows that the epidemic is merely delayed by the social distancing, and the final result is almost identical to the case where no social distancing was attempted.

Scenario 08, in which substantial social distancing, effectively “lockdown” (80% reduction in average contacts), is implemented for 1 month, followed by a relaxation of social distancing to approximately 50% of baseline levels results in only a small initial epidemic, which closely resembles the observed data in both magnitude and duration, with ongoing suppression, but not complete elimination, of cases following the relaxation of the lockdown period.

Scenario 09, which is the same as scenario 08 except that social distancing slowly relaxes all the way back to baseline levels, results in a “second wave,” which is much better than the first, but still only one-tenth the size of the no-intervention model epidemic.

Scenario 10 is the same as scenario 09 except that the isolation rate is increased postlockdown to double the level in the other scenarios. This simulates very high testing rates and very efficient case-based interventions. The result is almost complete suppression of any second or subsequent waves, despite social distancing slowly being relaxed to baseline levels.

### Supplementary Files

The outputs of all the simulations reported here are provided in Multimedia Appendix 1 and 2 in CSV (comma-separated values) format.

## Discussion

### Principal Results

COVOID allocates each member of its hypothetical population to one of seven compartments. The number of times individuals in the various compartments interact with each other and their probability of transmitting infection at each interaction can be varied to simulate the effects of interventions.

Using COVOID for March 30, 2020, we were able to replicate the epidemic response patterns to specific social distancing intervention scenarios reported by other investigators at that time and to further investigate emergence of herd immunity effects with even lower levels of social distancing. Importantly, we confirmed “second wave” rebound behaviors of the epidemic after the higher levels of social distancing were relaxed, a phenomenon that was not remarked upon in the study that motivated the COVOID model [5].

Using COVOID on April 30, 2020, the simulated incidence for three local areas of Sydney from March 1 to April 30, 2020, was similar to the actual, observed epidemic curve in two of the intervention scenarios that were modeled. These two scenarios (08 and 10) are also arguably closest to the interventions that took place in Sydney during the months of March and April 2020. At the time of writing (early May 2020), these two scenarios also point to possible postlockdown “exit strategy” futures in which social distancing is gradually relaxed over several months, either to intermediate levels compared to pre–COVID-19, or completely but, in the latter case, allied with greater expanded testing to detect cases as early as possible, and extremely efficient and swift isolation of cases and associated contact tracing and quarantining. At this stage, both Australian and NSW governments appear to be contemplating a path similar to scenario 10 and have invested heavily in both testing capacity and case-based intervention capacity, including deployment of a smartphone contact tracing “app” nationwide [19].

### Limitations

COVOID was developed quickly in a rapidly evolving environment in terms of our understanding of the infection dynamics of COVID-19, and thus, several key parameters had to be informed by expert opinion from colleagues and other heuristics. In addition, we could not test the effects of closures of schools or universities because COVOID is a global mixing model that does not reflect mixing in specific settings such as schools or workplaces.

The absence of age-specific parameters is another key limitation of the current model; although, in the absence of detailed data on age differences in COVID-19 disease progression, with the exception of death rates, the added complication of age-specificity may not add much. Future versions of COVOID, which will leverage the POLYMOD age-specific contact matrices [20], will use age as an attribute of each person in the simulation.

Agent-based models are notoriously computationally intensive, and the COVOID model is no exception, although it does take advantage of parallel computation available on almost all computers these days. However, computational burden means that it is impractical to simulate very large populations; although, the model was successfully trialed with populations of 1 million. Further work is underway to improve the processing efficiency by rewriting critical sections of the R code as C++.

It is beyond the scope of this paper to undertake a comprehensive comparison of agent-based computational models with the more commonly used continuous- or discrete-time mathematical models implemented as systems of ordinary differential equations (ODE). However, it is well recognized that the systems of equations needed by mathematical models that seek to simulate different, potentially conditional or contingent, behaviors in subgroups can quickly become unwieldy and difficult to define. Adding stochastic behavior, which may be particularly important for modeling “exit strategies” where small numbers of incident cases may (or may not) establish new transmission chains, is an additional task with ODE models, whereas it is intrinsic in most computational models.

Due to time constraints in the rapidly evolving situation in March 2020, the initial COVOID model was released as a set of R scripts rather than as a software package with detailed documentation or simple user interface, and hence, its potential user base was limited to modelers and researchers with relevant technical expertise. Our team and collaborators are currently developing an extended open-source COVOID package for R comprising of a suite of tools to explore intervention scenarios using several categories of models.

### Comparison With Prior Work

In our initial simulations for March 30, 2020, we explicitly sought to test the simulations produced by COVOID with those reported by Chang et al [5] based on a highly detailed agent-based models for the entire Australian population. Our findings regarding social distancing interventions with varying degrees of compliance are very similar to theirs [5] and broadly consistent with those for social distancing interventions produced by the UK Imperial College agent-based model [4]. Importantly, COVOID and the other agent-based models all highlight the potential for resurgence of cases once social distancing measures are relaxed. This indicates that these measures may “buy time” in which to put in place comprehensive measures for testing, case finding, isolation, and quarantine, rather than being sufficient in themselves to halt the epidemic.

It is encouraging that results produced by COVOID are similar to those so far reported from the more complex agent-based models that require highly detailed population data and high-performance computing.

As of April 30, 2020, we could locate only one other study that compared modeled predictions with observed data for COVID-19 incidence for a specific population. Turk et al [21] compared the DCM susceptible-infected-removed model predictions to observed prevalence data for North Carolina and the United States, and used EpiModel to simulate interventions by altering the probability of infection. They reported that a model incorporating parameters that simulated a stay-at-home intervention increasingly produced a better fit to the observed data as the epidemic progressed and emphasized the value of flexible, continuously iterated models for informing local responses.

### Conclusions

COVOID allows rapid modeling of many potential intervention scenarios, can be tailored to diverse settings, and requires only standard computing infrastructure. It replicates the epidemic response patterns produced by other models that require highly detailed population-level data, and its predicted epidemic curve was similar in form to that observed in Sydney, Australia. In answer to the call for transparency and reproducibility in COVID-19 models [22], it is freely available as a tool to support public health decision makers in the current COVID-19 crisis. Our team and collaborators are currently developing an extended open-source COVOID package comprising of a suite of tools to explore intervention scenarios using several categories of models.

## Appendix

Multimedia Appendix 1 Model outputs for baseline and scenarios 01-05.

Multimedia Appendix 2 Model outputs for baseline and scenarios 06-10.

## Abbreviations

A: infected and asymptomatic
COVID-19: coronavirus disease
COVOID: COVID-19 Open-Source Infection Dynamics
CPU: central processing unit
CSV: comma-separated values
DCM: deterministic compartmental model
E: exposed
F: deaths due to COVID-19
H: requires hospitalization
I: infected and infectious
ICM: individual contact model
ODE: ordinary differential equations
NSW: New South Wales
Q: isolated
SEIR: susceptible-exposed-infectious-recovered
